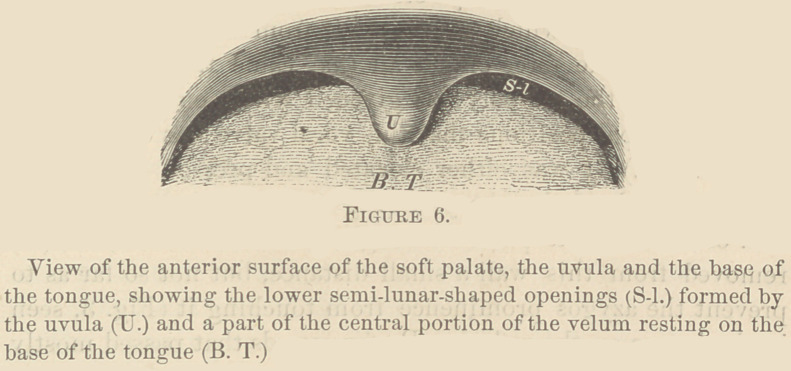# The Function of the Uvula and the Prominence Formed by the Azygos Uvulæ Muscles

**Published:** 1877-03

**Authors:** Thos. F. Rumbold

**Affiliations:** St. Louis, Mo.


### Selections.

### THE FUNCTION OF THE UVULA AND THE PROMINENCE FORMED BY THE AZYGOS UVULÆ MUSCLES.

By THOS. F. RUMBOLD, M.D., St. Louis, Mo.

(From the St. Louis Med. and Sur. Journal, Dec., 1876.)

In the spring of 1870 I had a patient whose right nostril was of sufficient caliber to admit my little finger in its whole length. The idea occurred to me at once that this case pre-

FIGURE 1.

Antero-posterior section of the head; R. reflector; S. P. soft palate; U. uvula; E. t. mouth of Eustachian tube; Ep. epiglottis.

sented an excellent opportunity for examining the uvula; and as our authorities say of this grape-shaped appendage, that

"its use is not clear,"* I determined to take advantage of this opportunity to inspect its motions during mastication, deglutition and vocalization.

I had the patient keep this nostril wide open with a Kramer bi-valve ear speculum. Through this large nasal passage, thus dilated, I passed a reflector reaching to the posterior wall of the pharyngo-nasal cavity (Fig. 1, R); on the mirror (R) I directed a calcium light, illuminating the parts under observation, so that the image was reflected back to my eye very distinctly.

FIGURE 2.

View of the posterior nasal passages, the posterior surface of the soft palate and base of the tongue; Pt N. posterior nares; E. t. Eustachian tubes; Az-Pr. azygos prominence, on the upper surface of the soft palate formed by the azygos uvulæ muscles; S-l. semi-lunar openings formed by the tongue, uvula and soft palate; T. base of tongue; Ep. epiglottis; U. uvula.

In this way I was enabled to inspect the upper or posterior surface of the soft palate, and the prominence or ridge on it that the azygos uvulæ form (Fig. 2, Az-Pr), the base of the tongue (T), the epiglottis (Ep), and the larynx, at the time of the attempted phonation of the sound "æ" with the mouth closed.

My observations on this patient were continued for a period of five weeks. Subsequently I made numerous observations

* Dunglinson's Med. Dic.

of a similar character on six other patients, each of whom had lost the septum nasi, but had perfect soft palates.

From notes that were taken at the time of these inspections — about seventy-five in number — I will state what part, in my judgment, the soft palate, the uvula and the azygos prominence (Fig. 2, Az-Pr., and Fig. 3) take in the acts of mastication and deglutition, and what were their positions at the time of the phonation of such simple sounds as show enough of their action to demonstrate their proper function; reserving for the

FIGURE 3.

The image, seen on the hinged reflector (R), of the lower edge of the soft palate and the lower or posterior concave surface of the uvula (U), showing, also, the higher semi-lunar-shaped openings (S-l) made by the azygos prominence touching the posterior wall of the pharynx.

near future the details concerning the position of these three organs, as well as that of the base of the tongue and the epiglottis during the phonation of specified sounds.

Although I know now that the uvula and the azygos prominence (Figs. 2 and 3) are not required to aid the acts of mastication and deglutition, yet I will give the results of the inspections while these processes were going on, because these results contain points of interest when taken in connection with phonation.

During mastication, the whole free border of the soft palate rested on the base of the tongue, reaching within a short distance of the epiglottis. In five of the cases, the uvula was not

FIGURE 4.

Antero-posterior section of the hard palate (hp.) and the soft palate (sp.), showing the position of the uvula resting on the base of the tongue (t.); Ep. epiglottis; E. t. mouth of Eustachian tube.

in sight at any time, and seemed to be doubled under the velum, so as to lie between it and the tongue (Fig. 4). Two patients had elongated uvulas, which sometimes hung down on the base of the tongue, and frequently touched the epiglottis. The uvula was always contracted; the evidence of this condition was the increased height of the azygos prominence, formed by the contracted azygos uvulæ (Fig. 2, Az-Pr).

During the act of deglutition, the soft palate was pushed backward by the alimentary bolus until the posterior wall of the pharynx was reached; the motion was continued in an upward direction until the upper surface of the velum was high enough to cover and close both Eustachian tubes (Fig. 2, S. P. E. t.) pushing the reflector (R) upward and forward; then the velum descended, as the alimentary bolus was swallowed, until its lower border touched the base of the tongue.

When I began to make observations, my attention was directed to the uvula alone; but the varying height of the azygos prominence during vocalization (Fig. 2, Az-Pr.) in this, my first patient, drew my attention to it, and what I discovered with respect to it was confirmed in the subsequent examination of the other cases, namely: that this prominence, whose existence I had known for some time, though I had never thought of assigning to it any function or use, was of as much importance in vocalization as, if not more, than the uvula itself; so that, while seeking for the function of this grape-shaped appendage, I discovered a new organ, and ascertained its function at the same time.

FIGURE 5.

Antero-posterior section of the hard palate (hp) and the soft palate (sp), showing the position of the velum closing the avenue to the pharyngo-nasal cavity; U. uvula; t. tongue: Ep. epiglottis.

During the vocalization of sounds that passed through the nose alone, the whole free border of the soft palate rested on the base of the tongue (Fig. 4), the uvula was not in sight at any time. During the vocalization of sounds that passed through the mouth alone, the soft palate was raised, and about 4‴ of its lower border was pressed against the posterior wall of the pharynx (Fig. 5).

From repeated inspections made while the velum was in each of these two positions, it appeared that all the sounds were uttered without the aid of either the uvula or the azygos prominence.

The favorable opportunity for observing what assistance is rendered by the azygos prominence and the uvula is during the phonation of such sounds as are required to pass through the mouth and nose at the same time. While these sounds were uttered, the soft palate was either suspended, so that but a

FIGURE 6.

View of the anterior surface of the soft palate, the uvula and the base of the tongue, showing the lower semi-lunar-shaped openings (S-l.) formed by the uvula (U.) and a part of the central portion of the velum resting on the base of the tongue (B. T.)

small part of its central portion of the uvula rested on the base of the tongue (Fig. 6), or it was raised to such a height that the azygos prominence touched the posterior wall of the pharynx (Fig. 3). In each situation that the velum occupied, the communication between the fauces and the mouth, and between the fauces and the pharyngo-nasal cavity, was divided into two equal, or nearly equal, semi-lunar openings. In the first position named, the division was made by the uvula and a small part of the central portion of the velum resting on the base of the tongue (Fig. 6, S-l), and in the second position the partition was made by the azygos prominence (Fig. 3, S-l), touching the posterior wall of the pharynx. In one patient I noticed, on several occasions, that the uvula seemed to be resting on the base of the tongue, while, at the smae time, the azygos prominence was touching the posterior wall of the pharynx.

The formation of the inferior or posterior surface of the uvula (Fig. 3, U,) as well as the peculiar position in which it hangs from the velum (Figs. 1 and 2, U,) indicates that this surface lies on the base of the tongue frequently, its extremity being directed forward, (Fig. 4). It is evident that this position is the best one in which it could be placed to prevent the free edge of the soft palate from being shaken by the force of the air from the lungs.

It was observed, repeatedly, that the free border of the velum was not at any time suspended in the current of air during vocalization, but was always situated in such positions that it received support, which prevented it from being thrown into vibrations by the force of the air that came from the larynx. To show how the support was given, I will mention again all of the principal positions that this vocal valve was observed to assume. (*a.*) It was either elevated and pressed against the posterior wall of the pharynx (Fig. 5, U,) during the phonation of sounds that passed through the mouth alone; or, (*b.*) removed from this wall a small distance, but not so far as to prevent the azygos prominence from touching it (Fig. 3, seen in the image on the reflector R,) for sounds that passed mostly through the mouth and a little through the pharyngo-nasal cavity; or, (*c.*) lowered to allow the uvula and a small part of the central part of the velum to rest on the base of the tongue (Fig 6,) for sounds that passed mostly through the nose and a little through the mouth; or, (*d.*) still lower, so that its whole free border rested on the base of the tongue (Fig. 4,) for the formation of sounds that passed the nose alone. In a few instances, as have been mentioned, I have seen the second and third positions combined, *i. e.*, the uvula resting on the base of the tongue, and the azygos prominence touching the posterior wall of the pharynx at the same time (Figs. 6 and 3).

From the effect of these positions of the velum on phonation, it would appear that one of its functions is to act as a valve, by directing the voice from the larynx into the mouth alone for the formation of one kind of tone; into the nose alone for another; and to divide the sound so as to allow it to escape from both of these openings for still others. It is evident that while the velum is resting wholly on the base of the tongue, or is pressed against the posterior wall of the pharynx, that the liability for its free border to vibrate by the force of the air is reduced to a minimum; but when this valve is in either position that requires it to divide the sound between the mouth and the nose, then, on account of its free edge being suspended and placed immediately in the current of air from the larynx, the liability for it to vibrate is increased to a maximum.

A provision is necessary to prevent these vibrations. This provision, I am led to believe from my observations, is found in the uvula and the azygos prominence formed by the azygos uvulæ muscles. It is located in the centre of this very mobile palate or valve, and by its support in both of the positions that require suspension (Figs 3 and 4,) prevents it from being shaken by the force of the current of air from the lungs. There can be no doubt, that if there were no uvula and azygos prominence to prevent this thin edge of suspended flesh from vibrating, it would be shaken to such a degree as to impart a tremulousness to the tone of all sounds forcibly uttered that pass through the mouth and nose at the same time.

The following questions have been asked frequently:

"1st. If the uvula is required to prevent the free border of the velum from vibrating during phonation, will not its loss impair the voice?

"2nd. How do you account for the improvement of the voice in many instances, after its removal?"

The excision of the uvula can affect those sounds only which are formed by its assistance, and not then, even, if they are pronounced with the usual strength of the voice, because the contact of the central portion of the velum on the base of the tongue will be support enough to prevent the velum from being shaken; therefore, the difficulty in pronouncing, in high and loud tones, those sounds that are required to pass mostly through the nose and a little through the mouth will be in proportion to the amount of loss of support that the velum suffers; as usual excisions leave a stump of the uvula and the central portion of the soft palate, these will prevent any vibrations during speech made with the *usual* force of the lungs.

I have observed that a patient, who has just undergone an operation for excision of an elongated and hypertrophied uvula, may talk immediately in an *ordinary* tone with greater ease than before the operation; but, just as soon as he utters words with *more* than the *usual* force of voice, such, for instance, as he would require to address a person across the street, some of the efforts will remind him of the excised uvula, and though not causing as much pain as the knife did, will cause so much that he will be compelled to cut his sentence short of its intended length. The reason of this effect on the uvula appears to me to be this: the heavy uvula had given so much support to the soft palate that, although it had been acting as an impediment to all kinds of sounds, the velum required very little of its own pressure on the base of the tongue (Fig. 6) to prevent it from being thrown into motion by the air from the larynx; but when the superabundant portion of the uvula was removed, the velum required greater pressure upon the base of the tongue to prevent these vibrations, and this pressure was the occasion of the pain. Of course, the loss of the whole of the uvula does not interfere with the formation of the two semi-lunar-shaped openings by the free border of the velum and the dorsum of the tongue (Fig. 6), by which the voice is allowed to escape from the mouth, and thus provide for perfect vocalization; it takes away a *part only* of the support from the soft palate. Even if there be no stump left by the excision, the tongue will learn to overcome the defect by the increased elevation of its dorsum, which may be made more convex than was required to form the two semi-lunar openings than when the whole of the uvula was present, and in this way allow both of a greater pressure and more of the central portion of the velum to rest on the tongue. But if the soft palate suffer so much of a loss of substance in its central portion that its concavity is equal to the convexity of the dorsum of the tongue, thereby preventing the formation of the semi-lunar-shaped openings, and neutralizing all support, there will be some sounds, such as pass mostly through the pharyngo-nasal cavity and a little through the mouth, given imperfectly, in spite of all efforts to overcome it, because the proper tone requires that the velum should be raised to allow a part of the sound to pass to the mouth, and this act of elevation exposes it to the force of the air from the larynx, which force is the cause of the imperfection of the sounds, by causing the unsupported edge to vibrate. Again, if the loss in the center of the velum be greater than can be closed by the greatest convexity of the dorsum of the tongue, the disability will be equal to that caused by a perforation of the soft palate, and in addition, there will be a tremulousness to many semi-nasal tones, on loud speaking, as addressing an individual at a distance. That the intermittent tone is occasioned by the vibrations of the central portion of the velum is evidenced by the pain in this part after lengthy speaking in a loud voice. This pain was experienced by two patients while under my care, whose soft palates were notched to this extent by ulceration.

In answer to the second question—" How to account for the improvement of the voice after the removal of the uvula?"—I would ask, if it is claimed that this improvement in speech is equal to the patient's vocalization at the time that his uvula was in a healthy condition. I am sure, because the observations made on this subject during the last five years have taught me to be so, that the answer to this question should be given in the negative. That a relative improvement in speech does follow an excision of an elongatated or hypertrophied uvula, there can be no doubt, because this operation brings the organ nearer to its normal size and condition; but it resembles the improvement made by perforating the membrana tympani in a case of deafness caused by a closure of the Eustachian tube; such an improvement can never equal the normal function of the organ. This being the case, the effect of the excision will be to remove the cause of a mechanical hindrance to every word uttered by the patient, made in any degree of force, and it will leave a stump which will not be a cause of hindrance, but a cause of an inability to pronounce some words on forced vocalization only, and this even will be overcome in time by the dorsum of the tongue becoming more convex. Therefore, to admit that the removal of a uvula thus diseased may improve the ability to speak in the usual tone of voice, does not prove that it was the uvula's removal that was the origin of the improvement, for, if such were the case, the excision of the healthy uvula would not only be advisable, but desirable.

The effect of the amputation of the whole of the uvula, besides its being a loss of the greater part of the support to the velum, prevents the formation of the azygos prominence to its greatest height, which is done by the contraction or elevation of the azygos uvulæ muscles, which terminates in the uvula. This height of the prominence is required to prevent, by its contact with the posterior wall of the pharynx, the vibrations of the velum during the formation of many semi-nasal sounds.

The nearer that the surgeon can make the diseased uvula take the shape and size of the normal one, the nearer will it approach its normal function; that is, rendering the soft palate a non-vibratory valve, which is required for perfect phonation.

## Figures and Tables

**Figure 1. f1:**
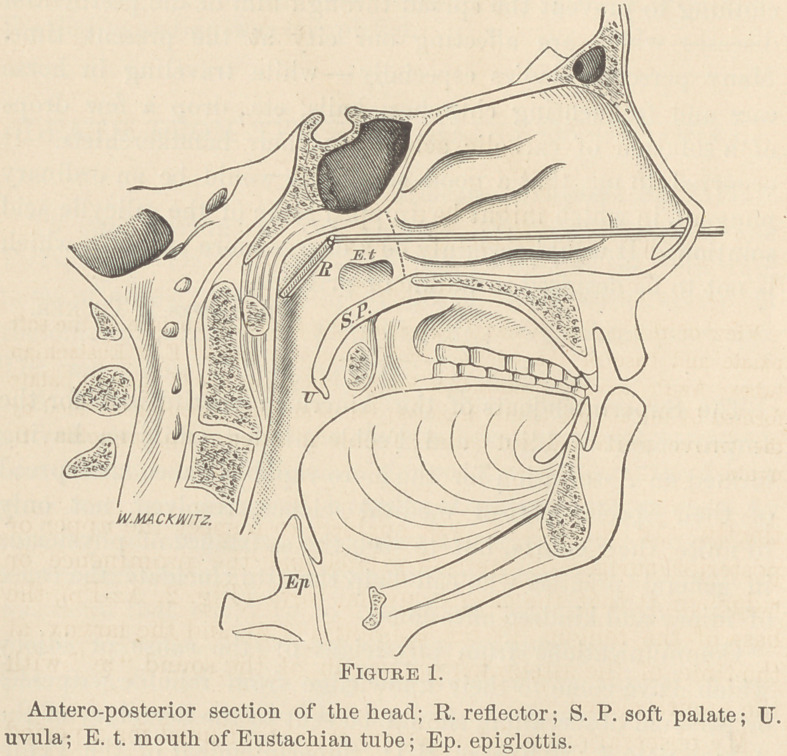


**Figure 2. f2:**
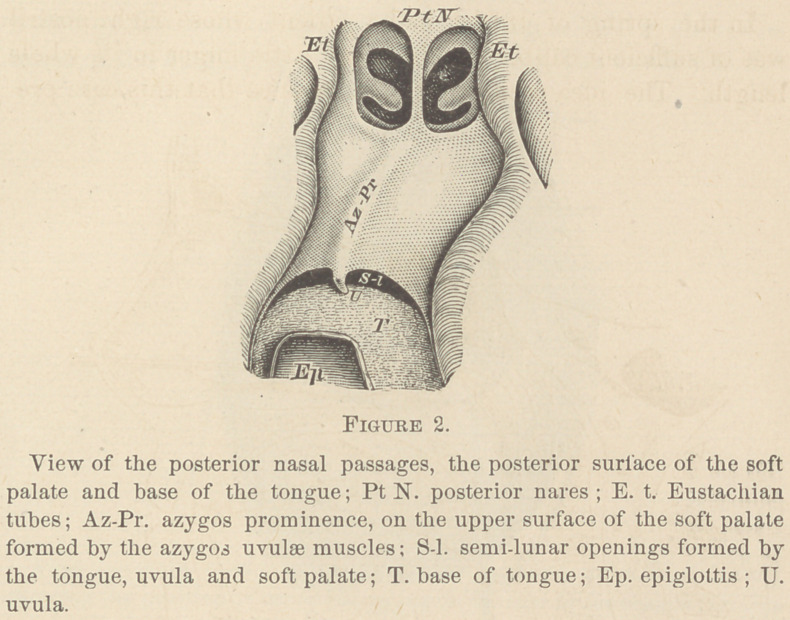


**Figure 3. f3:**
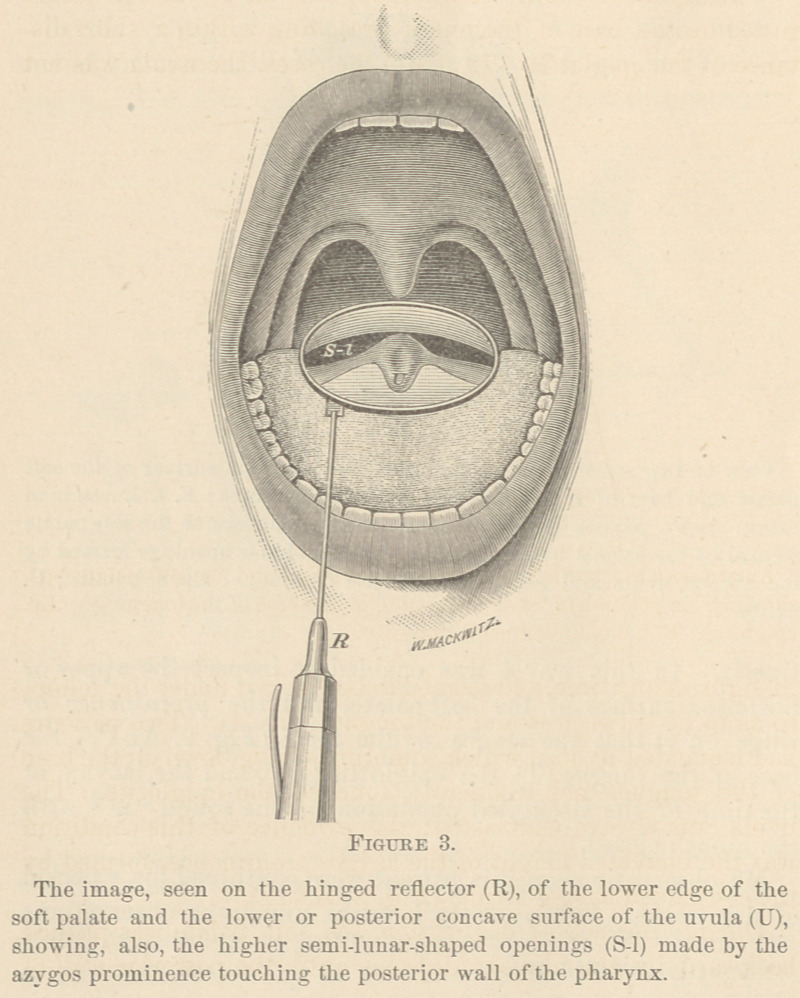


**Figure 4. f4:**
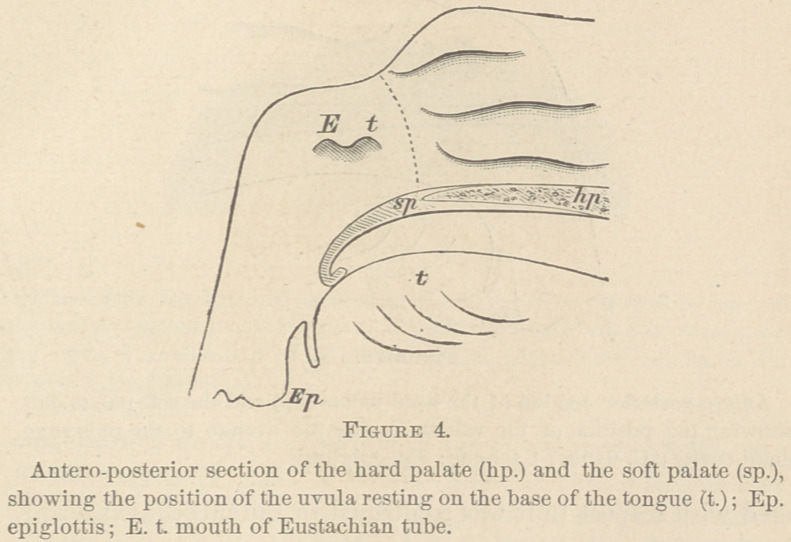


**Figure 5. f5:**
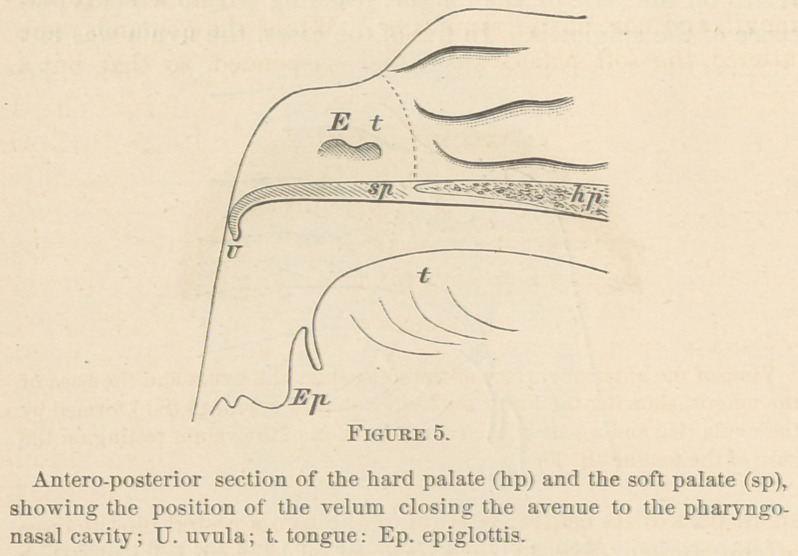


**Figure 6. f6:**